# Mitochondrial Dynamics in Diabetes Mellitus and Its Complications: Mechanisms, Pathological Roles, and Therapeutic Implications

**DOI:** 10.3390/ijms27198451

**Published:** 2026-09-22

**Authors:** Guohua Wang, Yige Zhao, Gaiting Liu, Yuhang Du, Shuchang Liu, Yang Yang, Sitong Bu, Baosheng Zhao

**Affiliations:** 1 Department of Pharmacology of Chinese Materia Medica, School of Chinese Materia Medica, Beijing University of Chinese Medicine, Beijing 102488, China; 17334054687@163.com (G.W.); zyg408716172@163.com (Y.Z.); gaiting2021@163.com (G.L.); 20230935235@bucm.edu.cn (Y.D.); 15647039408@163.com (S.L.); bucmyang2020@163.com (Y.Y.); colleen2003@163.com (S.B.); 2 Beijing Research Institute of Chinese Medicine, Beijing University of Chinese Medicine, Beijing 100029, China

**Keywords:** diabetes mellitus, diabetic complications, mitochondrial dynamics, therapeutic targets

## Abstract

Diabetes mellitus (DM) is a progressive metabolic disorder in which mitochondrial dysfunction is increasingly recognized as a central contributor to metabolic deterioration and diabetic complications. However, whether dysregulated mitochondrial dynamics represents a primary pathogenic driver, an adaptive response, or a consequence of metabolic stress remains incompletely understood. This review summarizes current advances in the molecular mechanisms governing mitochondrial fission, fusion, and mitophagy, with particular emphasis on their coordinated roles in mitochondrial quality control rather than isolated morphological changes. We discuss how disrupted mitochondrial dynamics contributes to pancreatic β-cell dysfunction, insulin resistance, diabetic nephropathy (DN), diabetic cardiomyopathy (DCM), diabetic retinopathy (DR), and diabetic osteoporosis (DOP) through context-dependent regulation of oxidative stress, bioenergetics, inflammation, and endoplasmic reticulum–mitochondria crosstalk. Furthermore, we critically evaluate emerging therapeutic strategies, including metformin, sodium–glucose cotransporter 2 inhibitors, mitochondrial fission inhibitors, and mitochondria-targeted antioxidants, highlighting both their therapeutic potential and current translational challenges. We propose that restoring the physiological equilibrium among mitochondrial fission, fusion, and mitophagy, rather than unidirectionally inhibiting a single pathway, represents a more promising strategy for preventing and treating diabetes and its complications.

## 1. Introduction

Diabetes mellitus (DM) is a chronic metabolic disorder characterized by persistent hyperglycemia resulting from impaired insulin secretion, insulin action, or both [1,2]. According to the International Diabetes Federation (IDF), the global prevalence of diabetes continues to rise rapidly, posing a major public health challenge worldwide [3]. Long-term hyperglycemia contributes to the development of multiple complications, including diabetic nephropathy (DN) [4], diabetic cardiomyopathy (DCM) [5], diabetic retinopathy (DR) [6], and diabetic osteoporosis (DOP) [7], which significantly increase morbidity, mortality, and healthcare burden. Despite substantial advances in glucose-lowering therapies, the incidence of diabetic complications remains high, indicating that the underlying pathogenic mechanisms are not fully understood.

Mitochondria are essential organelles responsible for cellular energy production, calcium homeostasis, reactive oxygen species (ROS) [8,9] signaling, and regulation of programmed cell death [10]. As the primary site of oxidative phosphorylation, mitochondria generate adenosine triphosphate (ATP) to meet cellular metabolic demands [11]. In addition to their bioenergetic functions, mitochondria act as important signaling hubs that regulate cellular adaptation to environmental and metabolic stress [12]. Mitochondrial dysfunction has been increasingly recognized as a central pathological feature of DM and its associated complications [13]. Hyperglycemia, lipid overload, and chronic inflammation can disrupt mitochondrial function, resulting in excessive ROS production, impaired ATP synthesis, mitochondrial DNA (mtDNA) damage, and activation of inflammatory and apoptotic pathways [14,15].

To maintain mitochondrial quality and functional integrity, mitochondria continuously undergo dynamic remodeling through coordinated processes of mitochondrial fission, fusion, and mitophagy, collectively referred to as mitochondrial dynamics [16]. Mitochondrial fission facilitates mitochondrial division and removal of damaged organelles, whereas mitochondrial fusion promotes the exchange of mitochondrial contents and preserves mitochondrial function [17]. Mitophagy selectively eliminates dysfunctional mitochondria to maintain cellular homeostasis [18]. Under physiological conditions, these processes remain tightly balanced. However, under diabetic conditions, excessive mitochondrial fission, impaired fusion, and defective mitophagy frequently occur, leading to mitochondrial fragmentation, oxidative stress, metabolic dysfunction, and cellular injury [19,20]. These abnormalities can further aggravate metabolic and cellular stress, thereby contributing to the progression of diabetes and promoting the development of diabetic complications through persistent mitochondrial dysfunction, oxidative stress, inflammation, and impaired cellular homeostasis [21].

Accumulating evidence has demonstrated that dysregulated mitochondrial dynamics plays a critical role in the pathogenesis of DM and its complications [22]. Alterations in key regulators of mitochondrial dynamics, including dynamin-related protein 1 (DRP1), mitochondrial fission factor (MFF), mitofusin 1 (MFN1), mitofusin 2 (MFN2), optic atrophy protein 1 (OPA1), PTEN-induced kinase 1 (PINK1), and Parkin, have been associated with pancreatic β-cell dysfunction, insulin resistance, renal injury, cardiac remodeling, retinal degeneration, and impaired bone metabolism [23,24,25].

Although numerous studies have investigated the relationship between mitochondrial dysfunction and diabetes, a comprehensive understanding of the molecular mechanisms linking mitochondrial dynamics to diabetic complications remains incomplete. Furthermore, recent advances in mitochondrial biology have identified several novel therapeutic targets that may improve mitochondrial homeostasis and mitigate disease progression. Therefore, this review summarizes current knowledge regarding the molecular mechanisms of mitochondrial dynamics and discusses their pathological roles in DM and DN, DCM, DR, and DOP. In addition, emerging therapeutic strategies targeting mitochondrial dynamics and future research directions are highlighted to provide new insights into the prevention and treatment of DM and its complications.

## 2. Molecular Mechanisms of Mitochondrial Dynamics

Mitochondrial dynamics is primarily regulated through three interconnected processes: mitochondrial fission, mitochondrial fusion, and mitophagy. These processes coordinate mitochondrial morphology, quality control, turnover, and cellular adaptation to metabolic stress. Mitochondrial fission and fusion dynamically regulate mitochondrial network architecture and facilitate the segregation and redistribution of mitochondrial components, whereas mitophagy selectively removes damaged or dysfunctional mitochondria to maintain mitochondrial quality and homeostasis. The key molecular regulators and mechanisms involved in mitochondrial fission, fusion, and mitophagy are summarized in Table 1, and their respective mechanisms are described in detail in the following sections.

### 2.1. Fission

Mitochondrial fission is a highly regulated process that divides a single mitochondrion into two daughter organelles and plays an essential role in mitochondrial quality control, organelle distribution, and cellular adaptation to metabolic stress [26,27]. Under physiological conditions, mitochondrial fission facilitates the segregation of damaged mitochondrial components and cooperates with mitophagy to maintain mitochondrial homeostasis [28]. However, excessive mitochondrial fission has been increasingly recognized as a key contributor to mitochondrial dysfunction in DM and its complications [29].

Mitochondrial fission is primarily regulated by DRP1 [30,31,32], MFF [33], and fission protein 1 (FIS1). DRP1 is a cytosolic GTPase that remains inactive in the cytoplasm under basal conditions. Upon activation, DRP1 is recruited to the outer mitochondrial membrane (OMM) through receptor proteins including MFF and FIS1, where it oligomerizes into ring-like structures surrounding mitochondria and mediates membrane constriction through GTP hydrolysis, ultimately leading to mitochondrial division [34]. The activity of DRP1 is tightly controlled by multiple post-translational modifications, including phosphorylation, SUMOylation, ubiquitination, and acetylation [10,11]. Under conditions of energetic stress, AMP-activated protein kinase (AMPK) phosphorylates MFF, thereby enhancing the interaction between MFF and DRP1 and promoting DRP1 recruitment to mitochondria [35]. In addition, several kinases, including cyclin-dependent kinase 1 (Cdk1), protein kinase Cδ (PKCδ), extracellular signal-regulated kinase (ERK), Ca2+/calmodulin-dependent protein kinase II (CaMKII), and Rho-associated coiled-coil-containing protein kinase 1 (ROCK1), can phosphorylate DRP1 and enhance its fission activity [36,37]. Besides phosphorylation, SUMOylation also plays an important role in regulating DRP1 stability and function [30,38]. SUMO-1 modification stabilizes DRP1 by preventing its lysosomal degradation and thereby promotes mitochondrial fission [39,40]. Conversely, SUMO-specific peptidase 3 (SENP3)-mediated deSUMOylation of SUMO-2/3 facilitates the interaction between DRP1 and MFF, further enhancing mitochondrial division [39,41]. In addition to phosphorylation and SUMOylation, acetylation and ubiquitination provide additional layers of regulation of DRP1 activity and stability. Under lipid overload, acetylation of DRP1 at lysine 642 (K642) promotes its phosphorylation at Ser616, oligomerization, mitochondrial translocation, and fission, linking metabolic stress to excessive mitochondrial fragmentation [42]. Acetylation-dependent regulation of DRP1 may therefore cooperate with phosphorylation to fine-tune mitochondrial fission under metabolic stress. Ubiquitination provides another important mechanism by regulating the turnover of DRP1 and other fission-related proteins. The mitochondrial E3 ubiquitin ligase MARCH5 regulates DRP1-dependent fission by controlling the ubiquitination and degradation of the DRP1 receptor MiD49, whereas Parkin-mediated ubiquitination promotes proteasomal degradation of DRP1 and thereby limits mitochondrial fragmentation [43]. Together, these post-translational modifications regulate the activity, localization, and stability of mitochondrial fission machinery and help determine the balance between physiological and pathological mitochondrial fragmentation.

Emerging evidence supports a context-dependent role of DRP1-mediated mitochondrial fission in diabetic pathophysiology rather than a simple pathogenic effect. Chronic hyperglycemia, oxidative stress, and lipid overload can enhance DRP1 activation and mitochondrial recruitment, promoting excessive fragmentation that is associated with impaired oxidative phosphorylation, altered ATP production, increased ROS generation, and apoptosis. Such abnormalities have been linked to pancreatic β-cell dysfunction, insulin resistance, DN, DCM, DR, and DOP. However, these associations should not be interpreted as evidence that DRP1 activation is uniformly detrimental, because basal and transient fission remains important for mitochondrial quality control, metabolic adaptation, and cellular function. Indeed, excessive suppression of mitochondrial fission can also impair mitochondrial metabolism and insulin secretion [44]. Therefore, the pathological consequence may depend more on the extent, duration, and cellular context of DRP1 activation than on DRP1 activity per se. From a therapeutic perspective, strategies aimed at selectively correcting excessive or sustained DRP1 activation, rather than completely blocking mitochondrial fission, may provide a more physiologically appropriate approach for restoring mitochondrial homeostasis in diabetes and its complications.

### 2.2. Mitochondrial Fusion

Mitochondrial fusion is a dynamic process through which individual mitochondria merge and exchange their mtDNA, proteins, lipids, and metabolites [45]. This process plays a crucial role in maintaining mitochondrial integrity, optimizing oxidative phosphorylation, and preserving cellular energy homeostasis. In contrast to mitochondrial fission, mitochondrial fusion enables the complementation of damaged mitochondrial components and contributes to mitochondrial quality control [46]. Impaired mitochondrial fusion has been increasingly recognized as an important contributor to mitochondrial dysfunction and metabolic disorders, including DM and its complications [47].

Mitochondrial fusion is coordinated by a group of large dynamin-related GTPases located on the outer and inner mitochondrial membranes (IMM), including MFN1, MFN2 [48], and OPA1. Fusion of the OMM is mediated by MFN1 and MFN2, whereas OPA1 regulates fusion of the inner mitochondrial membrane and maintains mitochondrial cristae structure [49]. Mitochondrial fusion is a highly coordinated multistep process involving the sequential fusion of the OMM and IMM. OMM fusion is primarily mediated by the mitofusins MFN1 and MFN2, which are dynamin-related GTPases located on the mitochondrial outer membrane. Upon mitochondrial tethering, MFN1 and MFN2 on adjacent mitochondria interact in trans and undergo GTP-dependent conformational changes that bring the two outer membranes into close proximity, ultimately promoting OMM fusion. Following OMM fusion, the IMM undergoes fusion through a distinct mechanism primarily mediated by OPA1, a dynamin-related GTPase localized to the inner mitochondrial membrane. OPA1 exists in long and short forms, and their coordinated activity is required for efficient IMM fusion and maintenance of cristae architecture. Thus, MFN1/MFN2-dependent OMM fusion and OPA1-mediated IMM fusion constitute sequential and functionally coupled steps that enable the complete fusion of mitochondria. Disruption of either step can impair mitochondrial network remodeling and compromise mitochondrial homeostasis [50].

MFN1 and MFN2 are transmembrane GTPases localized on the OMM and share significant structural similarity. Through homotypic and heterotypic interactions between adjacent mitochondria, MFN1 and MFN2 facilitate mitochondrial tethering and membrane fusion [51]. Among these proteins, MFN1 exhibits stronger GTPase activity and plays a dominant role in membrane fusion efficiency, whereas MFN2 possesses additional regulatory functions beyond mitochondrial fusion. In particular, MFN2 participates in the formation of mitochondria-associated membranes (MAMs), which serve as critical communication platforms between the endoplasmic reticulum and mitochondria and are involved in calcium signaling, lipid metabolism, and cellular stress responses [52].

Accumulating evidence indicates that reduced expression of MFN2 is closely associated with insulin resistance, impaired glucose metabolism, and mitochondrial dysfunction [53]. Hyperglycemia and lipid overload can suppress MFN2 expression, resulting in fragmented mitochondrial networks, decreased ATP production, and excessive ROS generation. Furthermore, MFN2 deficiency has been implicated in the development of DN, DCM, and DR, highlighting its important role in maintaining metabolic homeostasis under diabetic conditions [54].

OPA1 is a dynamin-related GTPase localized to the inner mitochondrial membrane and is essential for inner membrane fusion and cristae remodeling [55]. In addition to regulating mitochondrial morphology, OPA1 maintains mitochondrial respiratory efficiency by preserving cristae architecture and preventing the release of pro-apoptotic factors such as cytochrome c [56]. Alterations in OPA1 expression or processing can disrupt mitochondrial structure, impair oxidative phosphorylation, and increase susceptibility to apoptosis [57].

Under diabetic conditions, decreased OPA1 expression and abnormal mitochondrial cristae remodeling have been reported in multiple tissues, including the kidney, heart, retina, and skeletal system [58,59]. Reduced OPA1 activity contributes to mitochondrial fragmentation, oxidative stress, and cellular injury, thereby accelerating the progression of diabetic complications. Conversely, restoration of OPA1 expression has been shown to improve mitochondrial function and alleviate metabolic abnormalities in experimental diabetic models [60].

Collectively, mitochondrial fusion serves as a critical protective mechanism that preserves mitochondrial function and cellular homeostasis. Dysregulation of MFN1-, MFN2-, and OPA1-mediated fusion promotes mitochondrial fragmentation and metabolic dysfunction, thereby contributing to the pathogenesis of DM and its complications. Therefore, strategies aimed at restoring mitochondrial fusion may represent promising therapeutic approaches for diabetes-related disorders.

### 2.3. Mitophagy

Mitophagy is a selective form of autophagy responsible for the identification and removal of damaged, dysfunctional, or superfluous mitochondria. As an essential component of mitochondrial quality control, mitophagy maintains mitochondrial homeostasis by preventing the accumulation of defective mitochondria. Through continuous mitochondrial turnover, mitophagy preserves cellular bioenergetics, metabolic flexibility, and stress adaptation. Increasing evidence suggests that impaired mitophagy is a characteristic feature of DM and contributes significantly to the development of diabetic complications.

In mammalian cells, two major molecular mechanisms have been identified: the ubiquitin-dependent PINK1/Parkin pathway and the receptor-dependent mitophagy pathway, which are activated by distinct cellular stress signals.

#### 2.3.1. Ubiquitin-Dependent Mitophagy

Under physiological conditions, PINK1 is continuously imported into healthy mitochondria and rapidly degraded. However, when mitochondria become damaged and lose their membrane potential, PINK1 accumulates on the OMM and serves as a molecular sensor of mitochondrial dysfunction. Stabilized PINK1 phosphorylates both ubiquitin and the E3 ubiquitin ligase Parkin, thereby promoting Parkin translocation from the cytosol to damaged mitochondria [61]. Activated Parkin ubiquitinates numerous outer mitochondrial membrane (OMM) proteins, including MFN1/2, VDAC, and MIRO, generating polyubiquitin chains that are recognized by selective autophagy adaptors such as OPTN, NDP52, and p62. These adaptor proteins subsequently recruit LC3-positive autophagosomes, ultimately leading to lysosomal degradation of dysfunctional mitochondria [62].

Activation of the PINK1/Parkin pathway facilitates the selective elimination of dysfunctional mitochondria and protects cells from oxidative damage [63]. Numerous studies have demonstrated that suppression of PINK1/Parkin-mediated mitophagy results in mitochondrial dysfunction, excessive ROS generation, inflammation, and apoptosis. In diabetic conditions, chronic hyperglycemia and metabolic stress frequently impair PINK1/Parkin signaling, leading to the accumulation of damaged mitochondria and exacerbation of tissue injury [46,64]. Restoration of PINK1/Parkin-mediated mitophagy has therefore emerged as a promising therapeutic strategy for diabetes-related complications.

#### 2.3.2. Receptor-Dependent Mitophagy

In contrast, receptor-dependent mitophagy is initiated independently of ubiquitination and primarily occurs during hypoxia or metabolic stress. This pathway relies on mitochondrial outer membrane receptors, including BCL2 Interacting Protein 3 (BNIP3), and FUN14 Domain-Containing Protein 1 (FUNDC1), each of which contains a conserved LC3-interacting region (LIR) that directly binds LC3 proteins to initiate autophagosome formation [65].

##### BNIP3-Mediated Mitophagy

BNIP3 is a hypoxia-responsive mitophagy receptor located on the OMM and is considered a major downstream target of hypoxia-inducible factor-1α (HIF-1α). Under normoxic conditions, BNIP3 expression remains relatively low because the protein undergoes continuous ubiquitination and proteasomal degradation. During hypoxia or metabolic stress, stabilization of HIF-1α markedly enhances BNIP3 transcription, leading to rapid accumulation of BNIP3 on the mitochondrial surface [66,67].

Following its induction, BNIP3 forms stable homodimers through its transmembrane domain and exposes its N-terminal LC3-interacting region (LIR) to the cytoplasm. Phosphorylation of the LIR motif at Ser17 and Ser24 further increases the binding affinity between BNIP3 and LC3/GABARAP proteins, enabling the isolation membrane to recognize damaged mitochondria and initiate mitophagosome formation. Unlike ubiquitin-dependent mitophagy, BNIP3 functions as a direct molecular bridge connecting mitochondria with the autophagic machinery [68,69].

Under metabolic stress and hypoxic conditions, BNIP3 expression is markedly increased and contributes to mitochondrial quality control by facilitating mitochondrial clearance [67]. Emerging evidence suggests that BNIP3-mediated mitophagy exerts protective effects in DN and DCM by reducing oxidative stress and maintaining mitochondrial integrity [70]. However, excessive or prolonged BNIP3 activation may also promote cell death, indicating that precise regulation of BNIP3 activity is essential for maintaining cellular homeostasis [71].

##### FUNDC1-Mediated Mitophagy

FUNDC1 is another OMM receptor that predominantly mediates hypoxia-induced mitophagy through reversible phosphorylation rather than transcriptional activation. Its cytoplasmic N-terminus contains a conserved N-terminal cytosolic LIR motif (YxxL) which serves as the docking site for LC3. Under physiological conditions, casein kinase 2 (CK2) phosphorylates serine 13, while Src kinase phosphorylates tyrosine 18, preventing the interaction between FUNDC1 and LC3 and thereby suppressing mitophagy [72,73].

Upon hypoxia or mitochondrial stress, inhibition of CK2 and Src is accompanied by activation of the Phosphoglycerate Mutase Family Member 5 (PGAM5), which dephosphorylates serine13. Simultaneously, the autophagy kinase Unc-51 Like Autophagy Activating Kinase 1 (ULK1) phosphorylates serine17, dramatically increasing the affinity of FUNDC1 for LC3 [65]. Activated FUNDC1 subsequently recruits LC3-positive phagophores to damaged mitochondria, promoting selective mitochondrial sequestration and lysosomal degradation. This phosphorylation-dependent molecular switch allows FUNDC1 to rapidly respond to acute mitochondrial stress without requiring de novo protein synthesis [72,74].

Collectively, mitophagy represents a fundamental mitochondrial quality control mechanism that cooperates with mitochondrial fission and fusion to maintain cellular homeostasis. Dysregulation of PINK1/Parkin-, BNIP3-, and FUNDC1-mediated mitophagy contributes to mitochondrial dysfunction, oxidative stress, and metabolic abnormalities in DM. Therefore, therapeutic interventions aimed at restoring mitophagy may provide novel opportunities for preventing and treating diabetic complications. The major molecular mechanisms regulating mitochondrial dynamics and mitochondrial quality control are summarized in Figure 1.

## 3. Mitochondrial Dynamics in DM

DM is a complex metabolic disorder characterized by chronic hyperglycemia, dysregulated lipid metabolism, and chronic inflammation. Increasing evidence indicates that mitochondrial dysfunction is a central pathological feature of diabetes and contributes to metabolic deterioration [17]. However, the relationship between mitochondrial dynamics and mitochondrial dysfunction is not strictly unidirectional. Dysregulation of mitochondrial fission, fusion, and mitophagy can impair mitochondrial bioenergetics, redox homeostasis, calcium handling, and mitochondrial quality control, thereby contributing to mitochondrial dysfunction [20]. Conversely, diabetes-associated metabolic stress, including hyperglycemia, lipid overload, and oxidative stress, can directly impair mitochondrial function and subsequently alter the activity or expression of key regulators of mitochondrial dynamics, such as DRP1, MFN2, OPA1, PINK1, and Parkin [75,76]. Thus, mitochondrial dynamics and mitochondrial dysfunction may reinforce each other through a self-amplifying cycle rather than existing as a simple upstream–downstream relationship.

Under DM, excessive mitochondrial fission, impaired fusion, and defective mitophagy are frequently accompanied by reduced oxidative phosphorylation, excessive ROS production, altered calcium homeostasis, and impaired ATP metabolism [77]. These functional abnormalities can further activate stress-responsive pathways and exacerbate mitochondrial remodeling, thereby reinforcing the imbalance between fission, fusion, and mitochondrial turnover [78]. Therefore, mitochondrial dynamics should be considered both a mediator and a consequence of mitochondrial dysfunction during the development and progression of diabetes mellitus and its complications. Importantly, the pathological outcome depends on the coordinated balance among mitochondrial fission, fusion, and mitophagy rather than alterations in any single process alone.

Pancreatic β-cells and insulin-responsive tissues are particularly dependent on mitochondrial function because glucose-stimulated insulin secretion and cellular glucose utilization require adequate ATP production [53]. Under diabetic conditions, chronic hyperglycemia, lipotoxicity, oxidative stress, and inflammation disrupt mitochondrial dynamics, resulting in excessive mitochondrial fission, impaired fusion, and defective mitophagy [79,80]. These alterations not only impair insulin secretion but also promote insulin resistance and metabolic dysfunction in peripheral tissues.

Recent studies have demonstrated that key regulators of mitochondrial dynamics, including DRP1, MFN2, OPA1, PINK1, and Parkin, are significantly altered in diabetic tissues [81,82,83]. Dysregulation of these proteins contributes to β-cell dysfunction, impaired glucose homeostasis, and progressive metabolic deterioration. Consequently, mitochondrial dynamics has emerged as an important mechanistic link between metabolic stress and DM.

### 3.1. Mitochondrial Dynamics and β-Cell Dysfunction

Pancreatic β-cells play a central role in maintaining glucose homeostasis by sensing changes in blood glucose levels and secreting insulin accordingly. Because glucose-stimulated insulin secretion (GSIS) depends on mitochondrial oxidative phosphorylation, ATP generation, and coordinated metabolic signaling, β-cells are particularly sensitive to disturbances in mitochondrial homeostasis [80]. Under physiological conditions, a dynamic balance among mitochondrial fission, fusion, and mitophagy maintains mitochondrial morphology, bioenergetic efficiency, calcium homeostasis, and redox balance, thereby supporting appropriate insulin synthesis and secretion [84]. In diabetes, chronic hyperglycemia, lipotoxicity, inflammation, and oxidative stress disrupt this balance and progressively impair β-cell adaptation and survival [85].

Mitochondrial dynamics is essential for maintaining β-cell metabolic homeostasis and GSIS. Mitochondrial fission, primarily mediated by DRP1, facilitates mitochondrial turnover and metabolic adaptation, whereas fusion, mediated by MFN1, MFN2, and OPA1, supports mitochondrial content exchange, cristae organization, and oxidative phosphorylation. Under diabetic conditions, hyperglycemia and lipid overload promote DRP1 activation and mitochondrial recruitment, resulting in excessive mitochondrial fragmentation [86,87]. This pathological remodeling is accompanied by impaired oxidative phosphorylation, altered ATP production, increased mitochondrial ROS, and loss of mitochondrial membrane potential, thereby disrupting the metabolic coupling required for GSIS [88,89]. However, mitochondrial fission is not intrinsically detrimental. Genetic or pharmacological inhibition of DRP1-dependent fission can also impair mitochondrial metabolic signaling and GSIS, indicating that a basal level of mitochondrial fission is required for normal β-cell function and that complete suppression of fission may be equally detrimental [90].

Importantly, mitochondrial dynamics is closely interconnected with endoplasmic reticulum (ER) homeostasis, providing a critical mechanistic link between mitochondrial dysfunction and β-cell failure. Mitochondria and the ER communicate through MAMs, which coordinate calcium transfer, lipid metabolism, redox signaling, and cellular metabolic homeostasis [88]. Under metabolic stress, DRP1 is also recruited to the ER and participates in ER remodeling. In pancreatic β-cells exposed to fatty acid overload, increased DRP1 activity is associated with ER expansion, mitochondrial fragmentation, and proinsulin retention within the ER, whereas inhibition of DRP1 or activation of AMPK attenuates these abnormalities [89]. In parallel, MFN2 contributes to ER–mitochondrial coupling at MAMs, indicating that impaired mitochondrial fusion may disrupt calcium exchange and ER homeostasis in addition to altering mitochondrial morphology and bioenergetics [91]. Persistent disruption of ER–mitochondrial homeostasis can activate the unfolded protein response and ER stress, thereby impairing proinsulin folding and insulin biosynthesis and ultimately promoting β-cell dysfunction and apoptosis [92]. Consistent with this mechanism, constitutive DRP1 overexpression in MIN6 β-cells reduced cellular insulin content and increased the expression of ER stress-related genes, suggesting that excessive manipulation of mitochondrial fission may impair insulin biosynthesis through disruption of ER proteostasis [93]. Collectively, these findings indicate that mitochondrial dynamics and ER stress are functionally interconnected processes and that both excessive fission and impaired fusion can disturb ER–mitochondrial homeostasis, thereby contributing to β-cell dysfunction, compromise insulin biosynthesis and secretion, and accelerate β-cell failure.

Mitophagy constitutes a complementary quality-control mechanism that removes damaged mitochondria and prevents their accumulation within β-cells. The PINK1–Parkin pathway is one of the best-characterized mechanisms, but receptor- and lysosome-dependent pathways involving BNIP3, FUNDC1, TFEB, and related factors also contribute to mitochondrial turnover under metabolic stress. Recent studies indicate that mitophagy in β-cells is not simply a constitutive degradation process but an adaptive response to metabolic challenge. In particular, mitochondrial or metabolic stress can activate lysosomal calcium signaling and TFEB-dependent transcriptional programs, thereby increasing mitophagy and mitochondrial functional adaptation. β-cell-specific impairment of this adaptive response exacerbates mitochondrial dysfunction, β-cell failure, and glucose intolerance [94]. These observations also indicate that static measurements of LC3, PINK1, or Parkin alone may not adequately represent mitophagic flux and should be interpreted together with functional or flux-based evidence.

### 3.2. Mitochondrial Dynamics and Insulin Resistance

Insulin resistance is a hallmark of T2DM and represents a major pathogenic event preceding persistent hyperglycemia [95]. It is characterized by a diminished cellular response to insulin in metabolically active tissues, including skeletal muscle, liver, and adipose tissue, resulting in impaired glucose uptake, increased hepatic glucose production, and systemic metabolic dysfunction [96]. Accumulating evidence indicates that dysregulated mitochondrial dynamics is closely associated with the development of insulin resistance by disrupting mitochondrial homeostasis, energy metabolism, and insulin signaling pathways [78].

Under physiological conditions, balanced mitochondrial fission, fusion, and mitophagy maintain mitochondrial morphology and bioenergetic efficiency, thereby supporting normal insulin responsiveness [97]. However, chronic nutrient overload, hyperglycemia, elevated free fatty acids, and oxidative stress disturb mitochondrial dynamics, leading to excessive mitochondrial fragmentation and impaired mitochondrial quality control [98]. These alterations reduce oxidative phosphorylation efficiency, decrease ATP production, and increase ROS generation, ultimately contributing to metabolic dysfunction and insulin resistance [99].

Excessive activation of DRP1-mediated mitochondrial fission has been identified as a major contributor to insulin resistance [100]. Hyperglycemia and lipid accumulation promote DRP1 phosphorylation and mitochondrial recruitment, resulting in fragmented mitochondria and impaired respiratory function [101]. Excessive mitochondrial fragmentation further enhances ROS production, which activates stress-responsive kinases, including c-Jun N-terminal kinase (JNK), inhibitor of κB kinase β (IKKβ), and protein kinase C (PKC) [102]. These kinases promote serine phosphorylation of insulin receptor substrate-1 (IRS-1), thereby suppressing phosphatidylinositol 3-kinase (PI3K)/protein kinase B (AKT) signaling and reducing insulin sensitivity [103]. Consequently, glucose transporter type 4 (GLUT4) translocation to the plasma membrane is impaired, leading to decreased glucose uptake in skeletal muscle and adipose tissue [104].

In contrast, mitochondrial fusion preserves mitochondrial integrity and metabolic flexibility by facilitating the exchange of mitochondrial contents and maintaining efficient oxidative phosphorylation [105]. MFN2, a key mediator of mitochondrial fusion, is consistently downregulated in insulin-resistant skeletal muscle and liver tissues [106]. Reduced MFN2 expression disrupts mitochondrial networking, impairs ATP synthesis, and exacerbates oxidative stress [107]. In addition to its role in mitochondrial fusion, MFN2 regulates MAMs, which are essential for calcium signaling, lipid metabolism, and insulin signaling [108]. Impaired MFN2-mediated ER–mitochondria communication has therefore been recognized as an important mechanism contributing to insulin resistance [107].

Mitophagy also plays a crucial role in maintaining insulin sensitivity by selectively removing dysfunctional mitochondria [109]. Under diabetic conditions, prolonged metabolic stress frequently suppresses PINK1/Parkin-mediated mitophagy, resulting in the accumulation of damaged mitochondria and sustained oxidative stress [110]. Defective mitochondrial clearance further aggravates inflammatory responses through activation of the NLRP3 inflammasome and nuclear factor-κB (NF-κB) signaling, thereby creating a vicious cycle that exacerbates insulin resistance [111]. Conversely, restoration of mitophagy has been shown to improve mitochondrial quality, reduce ROS production, and partially restore insulin responsiveness in experimental diabetic models [112].

Recent studies have further demonstrated that mitochondrial dynamics is closely integrated with lipid metabolism and chronic low-grade inflammation, two major drivers of insulin resistance [113]. Excessive intracellular lipid accumulation promotes mitochondrial dysfunction and oxidative stress, whereas mitochondrial ROS amplifies inflammatory cytokine production, including tumor necrosis factor-α (TNF-α), interleukin-6 (IL-6), and interleukin-1β (IL-1β). These inflammatory mediators further impair insulin signaling, thereby accelerating metabolic deterioration [114]. Therefore, dysregulated mitochondrial dynamics not only disrupts cellular energy metabolism but also links oxidative stress, inflammation, and impaired insulin signaling during the progression of T2DM [115].

Collectively, excessive mitochondrial fission, impaired mitochondrial fusion, and defective mitophagy cooperatively contribute to mitochondrial dysfunction and insulin resistance under diabetic conditions. Restoration of mitochondrial dynamics has therefore emerged as a promising therapeutic strategy for improving insulin sensitivity, preserving metabolic homeostasis, and delaying the progression of T2DM.

## 4. Mitochondrial Dynamics in Diabetic Complications

Diabetic complications are the major causes of disability and mortality in patients with DM and involve multiple organs, including the kidney, heart, retina, and skeletal system. As illustrated in Figure 2, DN, DCM, DR, and DOP represent the major organ-specific complications associated with mitochondrial dysfunction and are discussed in the following sections. Although these complications exhibit distinct pathological characteristics, they share several common mechanisms, such as chronic hyperglycemia, oxidative stress, inflammation, mitochondrial dysfunction, and metabolic imbalance. Increasing evidence indicates that dysregulated mitochondrial dynamics is a central event linking metabolic stress to tissue injury in diabetic complications.

Under diabetic conditions, persistent hyperglycemia and lipid overload disrupt the balance between mitochondrial fission, fusion, and mitophagy, resulting in mitochondrial fragmentation, impaired oxidative phosphorylation, excessive ROS production, and defective mitochondrial quality control [116]. These alterations trigger inflammatory responses, apoptosis, fibrosis, and metabolic reprogramming, ultimately contributing to progressive organ dysfunction [117]. Importantly, the regulatory mechanisms of mitochondrial dynamics vary among different tissues, reflecting the distinct metabolic demands and pathological features of individual diabetic complications [118].

Recent studies have demonstrated that key regulators of mitochondrial dynamics, including DRP1, MFN2, OPA1, PINK1, and Parkin, are significantly altered in DN, DCM, DR, and DOP [119]. These molecules not only govern mitochondrial morphology and quality control but also regulate oxidative stress, inflammatory signaling, apoptosis, and cellular metabolism [120]. Therefore, targeting mitochondrial dynamics has emerged as a promising therapeutic strategy for preventing tissue injury and delaying the progression of diabetic complications [121]. The following sections summarize the current understanding of mitochondrial dynamics in major diabetic complications and highlight the underlying molecular mechanisms involved.

### 4.1. Mechanisms of Mitochondrial Dynamics in DN

DN is one of the most common microvascular complications of DM and remains the leading cause of chronic kidney disease and end-stage renal disease worldwide. Persistent hyperglycemia induces metabolic disturbances, oxidative stress, inflammation, and mitochondrial dysfunction, all of which contribute to progressive renal injury [122]. Although mitochondrial fragmentation is frequently observed in diabetic kidneys, accumulating evidence suggests that altered mitochondrial morphology should not be interpreted as an isolated pathogenic event. Instead, mitochondrial remodeling represents a dynamic response to persistent metabolic stress, and its functional consequences depend on the balance between mitochondrial fission, fusion, and mitophagy [123].

Renal tubular epithelial cells and podocytes are highly dependent on mitochondrial oxidative phosphorylation to meet their substantial energy demands. Under diabetic conditions, chronic hyperglycemia and lipid overload disrupt the balance between mitochondrial fission, fusion, and mitophagy, resulting in excessive mitochondrial fragmentation, impaired ATP production, and increased ROS generation. Accumulated ROS further aggravates mtDNA damage, activates inflammatory signaling pathways, and promotes apoptosis and renal fibrosis, thereby accelerating the progression of DN [121,122].

Excessive mitochondrial fission, primarily mediated by DRP1, is a characteristic feature of diabetic kidneys. Hyperglycemia stimulates DRP1 phosphorylation and mitochondrial translocation, leading to mitochondrial fragmentation and loss of mitochondrial membrane potential. Inhibition of DRP1 has been shown to reduce oxidative stress, preserve mitochondrial function, and attenuate podocyte injury and renal fibrosis in experimental diabetic models, highlighting excessive mitochondrial fission as a key pathogenic mechanism in DN [124]. Mitochondrial fusion is markedly impaired during DN. Reduced expression of MFN2 and OPA1 disrupts mitochondrial network integrity, compromises oxidative phosphorylation, and increases susceptibility to apoptosis. In addition to regulating mitochondrial fusion, MFN2 also participates in endoplasmic reticulum–mitochondria communication through MAMs, thereby influencing calcium homeostasis and cellular metabolism. Restoration of mitochondrial fusion has therefore been associated with improved mitochondrial bioenergetics and reduced renal injury. Conversely, persistent mitochondrial hyperfusion and the formation of excessively enlarged mitochondria have also been implicated in DN progression. Although mitochondrial fusion is generally protective under physiological conditions, uncontrolled hyperfusion impairs mitochondrial turnover, promotes the accumulation of dysfunctional mitochondria, increases ROS production, and ultimately aggravates podocyte injury and renal fibrosis, suggesting that both excessive fission and excessive fusion contribute to diabetic renal injury through disruption of mitochondrial dynamic homeostasis [123].

Mitophagy serves as another essential mitochondrial quality control mechanism in diabetic kidneys. The PINK1/Parkin pathway selectively removes damaged mitochondria and limits ROS accumulation under physiological conditions [125]. However, persistent metabolic stress frequently impairs mitophagy in DN, leading to the accumulation of dysfunctional mitochondria and amplification of oxidative stress and inflammatory responses [126]. Besides the canonical PINK1/Parkin pathway, receptor-mediated mitophagy involving BNIP3 and FUNDC1 has also been implicated in renal protection by maintaining mitochondrial integrity and reducing tubular injury [127]. Nevertheless, excessive activation of mitophagy under certain pathological conditions may also contribute to cell death, suggesting that maintaining an appropriate level of mitochondrial turnover is essential for renal homeostasis.

Emerging evidence suggests that mitochondrial dynamics in DN should be interpreted as a homeostatic regulatory network rather than a simple imbalance between fission and fusion. Although excessive DRP1-dependent mitochondrial fission contributes to oxidative stress, podocyte injury, and renal fibrosis, physiological mitochondrial fission remains indispensable for mitochondrial quality control. Moderate DRP1 activity facilitates the segregation of damaged mitochondrial components and serves as a prerequisite for efficient mitophagy, including Parkin-independent pathways. Conversely, complete suppression of DRP1 may impair mitophagic flux and promote the accumulation of dysfunctional mitochondria, ultimately exacerbating mitochondrial dysfunction [128].

The biological roles of mitochondrial fusion are equally complex. While MFN1/2- and OPA1-mediated fusion generally preserves mitochondrial integrity and ATP production, persistent mitochondrial hyperfusion may inhibit mitochondrial turnover and disturb oxidative phosphorylation, indicating that the effects of fusion are highly context-dependent and influenced by disease stage, renal cell type, and metabolic status [129].

Therefore, future therapeutic strategies should aim to restore the physiological equilibrium among mitochondrial fission, fusion, and mitophagy, rather than unidirectionally inhibiting or enhancing a single pathway. Cell type-specific modulation of mitochondrial dynamics may provide a more precise approach for preventing DN progression while preserving renal bioenergetic homeostasis.

### 4.2. Mitochondrial Dynamics in DCM

DCM is a distinct cardiac complication of DM characterized by ventricular remodeling, myocardial fibrosis, impaired cardiac metabolism, and progressive cardiac dysfunction independent of hypertension or coronary artery disease [130]. Increasing evidence suggests that mitochondrial dysfunction is a central feature of DCM, as cardiomyocytes rely heavily on mitochondrial oxidative phosphorylation to sustain their high energy demands [131]. Among the various mechanisms contributing to mitochondrial dysfunction, dysregulated mitochondrial dynamics has emerged as a critical driver of myocardial injury under diabetic conditions.

Persistent hyperglycemia, lipotoxicity, and oxidative stress disrupt the balance between mitochondrial fission and fusion, leading to excessive mitochondrial fragmentation and impaired mitochondrial quality control. These alterations compromise oxidative phosphorylation, reduce ATP production, and promote excessive ROS generation, thereby impairing myocardial contractility and accelerating pathological cardiac remodeling [132].

Excessive activation of DRP1-mediated mitochondrial fission is consistently observed in experimental and clinical models of DCM. Hyperglycemia promotes DRP1 phosphorylation and mitochondrial translocation, resulting in fragmented mitochondria, loss of mitochondrial membrane potential, and enhanced ROS production [133]. Increased mitochondrial fission further activates apoptotic pathways and inflammatory responses, contributing to cardiomyocyte death, myocardial fibrosis, and ventricular dysfunction. Pharmacological inhibition of DRP1 has been shown to preserve mitochondrial morphology, attenuate oxidative stress, and improve cardiac function in diabetic animal models, highlighting mitochondrial fission as an important therapeutic target [134]. Nevertheless, physiological mitochondrial fission is also essential for mitochondrial renewal and quality control in cardiomyocytes. DRP1-mediated fission enables the segregation of damaged mitochondrial components and facilitates subsequent mitophagy, thereby maintaining mitochondrial homeostasis in the healthy myocardium [135]. This distinction may explain why complete pharmacological inhibition of DRP1 produces inconsistent outcomes among experimental studies. Moderate suppression of pathological fission appears cardioprotective by reducing mitochondrial fragmentation and oxidative stress, whereas excessive inhibition may impair mitophagic turnover, promote the accumulation of dysfunctional mitochondria, and ultimately reduce myocardial adaptability to metabolic stress [44].

Mitochondrial fusion also plays a protective role in maintaining cardiac mitochondrial homeostasis. Reduced expression of MFN2 and OPA1 has been associated with impaired mitochondrial networking, defective cristae organization, and diminished respiratory efficiency in diabetic hearts [136]. In addition to regulating mitochondrial morphology, MFN2 facilitates communication between the endoplasmic reticulum and mitochondria through MAMs, thereby influencing calcium homeostasis, energy metabolism, and cardiomyocyte survival [137]. Restoration of mitochondrial fusion has been reported to improve mitochondrial bioenergetics and reduce myocardial injury in experimental DCM.

Mitophagy represents another indispensable component of mitochondrial quality control in the diabetic heart. Under physiological conditions, PINK1/Parkin-mediated mitophagy selectively eliminates damaged mitochondria and maintains mitochondrial homeostasis [138]. However, chronic metabolic stress frequently impairs mitophagy during DCM progression, resulting in the accumulation of dysfunctional mitochondria, excessive ROS production, and persistent inflammatory activation. In addition to the canonical PINK1/Parkin pathway, receptor-mediated mitophagy involving BNIP3, BNIP3L/NIX, and FUNDC1 also contributes to mitochondrial quality control in cardiomyocytes [120]. Maintaining an appropriate level of mitophagy appears essential for preserving myocardial function, whereas either insufficient or excessive mitophagy may aggravate cardiac injury.

Recent studies have demonstrated extensive crosstalk between mitochondrial dynamics and signaling pathways involved in oxidative stress, inflammation, apoptosis, ferroptosis, and cardiac fibrosis. Dysregulated mitochondrial dynamics therefore not only impairs mitochondrial bioenergetics but also accelerates pathological remodeling of the diabetic heart [138]. Consequently, therapeutic strategies targeting mitochondrial dynamics have attracted considerable attention as potential approaches for preserving cardiac function and slowing the progression of DCM [120].

Collectively, these findings highlight mitochondrial dynamics as a pivotal determinant of cardiomyocyte survival and cardiac remodeling. Therapeutic interventions targeting mitochondrial fission–fusion balance may therefore provide novel approaches for preventing diabetic cardiac dysfunction.

### 4.3. Mechanisms of Mitochondrial Dynamics in DR

DR is a major microvascular complication of diabetes characterized by progressive retinal neurovascular dysfunction and remains a leading cause of vision loss worldwide. Growing evidence indicates that mitochondrial dynamic imbalance is an early pathogenic event contributing to retinal injury. Under hyperglycemic conditions, excessive mitochondrial fission accompanied by impaired fusion disrupts mitochondrial homeostasis, resulting in mitochondrial dysfunction, increased ROS production, and apoptosis of retinal vascular endothelial cells and pericytes, which precede the onset of clinically detectable DR [139].

Mitochondrial dysfunction also amplifies retinal inflammation through multiple mechanisms. Hyperglycemia-induced oxidative stress promotes the advanced glycation end products–receptor for advanced glycation end products (AGE–RAGE) signaling pathway and mitochondrial ROS accumulation, which activate macrophages, microglia, and the NLRP3 inflammasome, thereby enhancing the production of pro-inflammatory cytokines and accelerating retinal damage. Consistently, integrative transcriptomic analyses have identified mitochondrial function- and macrophage polarization-related genes as key regulators of DR progression, further supporting the close interplay between mitochondrial homeostasis and retinal inflammation [140].

In addition to regulating mitochondrial morphology, mitochondrial dynamics are tightly coupled to mitophagy. Hyperglycemia suppresses SIRT1 activity, leading to increased acetylation of MFN2 and impaired mitochondrial fusion, which promotes excessive mitochondrial fragmentation [139]. Impaired clearance of damaged mitochondria further aggravates oxidative stress and inflammatory responses, establishing a vicious cycle that accelerates retinal degeneration [140]. Conversely, restoration of MFN2 function or inhibition of its acetylation improves mitophagy and mitochondrial quality control. Receptor-mediated mitophagy may alleviate retinal injury by selectively removing dysfunctional mitochondria. However, excessive mitochondrial elimination could also impair neuronal bioenergetics. Consequently, therapeutic interventions targeting mitochondrial dynamics should preserve neuronal mitochondrial function while simultaneously limiting pathological inflammation [141], highlighting mitochondrial dynamics as a promising therapeutic target for preventing DR progression. Overall, dysregulated mitochondrial dynamics contribute to retinal neurovascular injury through coordinated regulation of oxidative stress, inflammation, and mitophagy. Targeting mitochondrial quality control may therefore represent a promising strategy for slowing DR progression [142].

### 4.4. Mechanisms of Mitochondrial Dynamics in DOP

DOP is a chronic skeletal complication of diabetes characterized by impaired bone formation, reduced bone mineral density, and increased fracture risk. Emerging evidence suggests that mitochondrial dynamic imbalance contributes substantially to osteoblast dysfunction and defective bone remodeling under diabetic conditions [143]. Hyperglycemia and AGEs disrupt mitochondrial homeostasis, leading to excessive ROS production, impaired osteoblast differentiation, and defective bone mineralization.

Mitophagy represents a critical component of mitochondrial quality control in osteoblasts. Physiological mitophagy removes dysfunctional mitochondria and maintains cellular energy metabolism, whereas dysregulated mitophagy contributes to mitochondrial dysfunction and osteoblast injury [144]. Oxidative stress further links mitochondrial dysfunction to diabetic bone loss. Advanced oxidation protein products (AOPPs) induce mitochondrial ROS accumulation, mitochondrial membrane depolarization, and intrinsic apoptotic signaling, ultimately promoting osteoblast apoptosis and bone microstructural deterioration. Activation of PINK1/Parkin-mediated mitophagy facilitates the clearance of damaged mitochondria and excessive ROS, thereby reducing apoptosis and partially restoring bone mass and mineral density, highlighting the protective role of mitochondrial quality control in DOP [145]. Recent studies have identified several regulators linking mitochondrial quality control to osteogenesis. Sirtuin 3 (SIRT3) promotes the deacetylation of forkhead box O3 (FOXO3), thereby facilitating its nuclear translocation and transcriptional activity. Nuclear FOXO3 directly binds to the promoter region of *Prkn* and enhances *Prkn* transcription, thereby increasing Parkin availability and promoting PINK1/Parkin-dependent mitophagy in osteoblasts. In diabetic osteoblasts, reduced SIRT3 expression leads to FOXO3 hyperacetylation and impaired nuclear translocation, consequently decreasing FOXO3 recruitment to the Prkn promoter and suppressing *Prkn* transcription. This impairment of the SIRT3–FOXO3–PINK1/Parkin axis compromises mitochondrial quality control and contributes to defective osteoblast function and bone formation. Conversely, restoration of SIRT3 activity promotes FOXO3 deacetylation and nuclear accumulation, restores Prkn transcription and PINK1/Parkin-mediated mitophagy, and consequently alleviates osteogenic impairment and bone loss in diabetes [146]. Nevertheless, hyperglycemia-induced AGE accumulation and oxidative stress impair mitochondrial BK channel expression, resulting in enhanced MAM and mitochondrial Ca^2+^ overload. Excessive mitochondrial Ca^2+^ subsequently activates the PINK1/Parkin pathway, leading to pathological mitophagy and osteoblast injury. Restoration of BK channel activity preserves mitochondrial Ca^2+^ homeostasis and prevents excessive mitophagic activation [147]. Importantly, this study highlights the cell type-specific regulation of autophagy in DOP by demonstrating that hyperglycemia suppresses AMPK-dependent autophagy in osteoblasts while simultaneously enhancing autophagy in osteoclasts. This finding suggests that mitochondrial quality control should be interpreted within the context of bone remodeling rather than as a uniform response to hyperglycemia. Interestingly, this opposite response indicates that identical metabolic stress may trigger distinct mitochondrial quality control programs in different bone cell types, reflecting the context-dependent nature of AMPK signaling during diabetic bone remodeling. However, several limitations should be considered. First, the in vivo experiments were performed using whole bone tissue, making it difficult to distinguish whether the observed changes in AMPK activity and autophagy originated from osteoblasts, osteoclasts, or other bone-resident cells. Second, the opposite activation patterns of AMPK were primarily demonstrated in vitro, and the upstream mechanisms responsible for this cell type-specific regulation remain unclear [148]. Therefore, future studies combining cell-specific genetic models with lineage-tracing approaches are needed to elucidate how mitochondrial dynamics and autophagy are differentially coordinated in osteoblasts and osteoclasts during diabetic bone remodeling.

Therapeutic modulation of mitochondrial fission appears to be context-dependent rather than uniformly beneficial through DRP1 inhibition. Pharmacological blockade of DRP1 protects osteoblasts from oxidative stress-induced dysfunction [149], whereas Zn/Sr-functionalized implants suppress excessive DRP1 expression and restore mitochondrial function under hyperglycemic conditions [150]. Conversely, siliconized collagen scaffolds can promote Drp1–Mff-mediated physiological fission while limiting pathological Drp1–Fis1 signaling [151], thereby enhancing mitochondrial quality and diabetic bone regeneration. However, mitochondrial dynamics in DOP are not always characterized by excessive mitochondrial fission. Hyperglycemia has been shown to reduce DRP1 expression and shift mitochondria toward a more fused morphology, accompanied by impaired mitochondrial biogenesis and reduced osteoblast migration and chemotaxis, ultimately contributing to defective bone remodeling [152]. Importantly, physiological mitochondrial fission is also required for effective mitophagy in osteoblasts. Hyperglycemia suppresses the PINK1/Drp1 signaling pathway, resulting in impaired mitophagic flux, accumulation of dysfunctional mitochondria, and defective osteoblast differentiation. Restoration of PINK1/Drp1-mediated mitophagy improves mitochondrial quality control and promotes osteoblastogenesis, suggesting that balanced mitochondrial fission is essential for bone homeostasis in DOP [144]. Collectively, these findings suggest that targeting mitochondrial dynamics and mitophagy represents a promising therapeutic strategy for preventing diabetes-associated bone deterioration.

Taken together, the available evidence suggests that the major unresolved issue in DOP is not whether mitochondrial dynamics or mitophagy are activated or suppressed, but how mitochondrial quality control becomes temporally and spatially uncoupled from the metabolic demands of bone cells. Most current studies infer mitochondrial dysfunction from changes in individual proteins, mitochondrial morphology, or endpoint measurements of mitophagy. However, these static readouts cannot adequately distinguish whether a change in DRP1, PINK1/Parkin, or autophagy reflects an adaptive response to mitochondrial stress, a compensatory mechanism that has become insufficient, or a maladaptive state that directly drives cellular injury. This distinction is particularly important in DOP, where mitochondrial remodeling must simultaneously accommodate osteoblast differentiation, osteoclast activity, energy demand, Ca^2+^ signaling, and oxidative stress. Thus, apparently contradictory observations across studies may not necessarily represent biological inconsistency, but rather different stages or cellular contexts of the same mitochondrial stress response.

A further conceptual limitation is that mitochondrial quality control is often investigated as isolated pathways, whereas fission, fusion, mitophagy, biogenesis, and metabolic adaptation are mechanistically interdependent processes. For example, mitochondrial fission can generate daughter mitochondria for selective removal, whereas mitochondrial biogenesis must subsequently replenish the mitochondrial pool; disrupting either side of this cycle may therefore produce a similar phenotype despite opposite changes in individual molecular markers. Likewise, enhancement of mitophagy without sufficient mitochondrial biogenesis may deplete functional mitochondria, whereas excessive mitochondrial biogenesis in the presence of defective quality control may expand the pool of damaged organelles. From this perspective, mitochondrial dysfunction in DOP may be better conceptualized as a failure of mitochondrial quality-control coordination rather than an abnormality of any single pathway.

This conceptual shift has important implications for therapeutic development. Rather than pursuing universal inhibition of DRP1 or indiscriminate activation of mitophagy, future interventions should aim to restore the coupling between mitochondrial remodeling, selective mitochondrial clearance, and mitochondrial renewal in a cell- and disease-stage-specific manner. Such a strategy will require moving beyond conventional endpoint measurements toward dynamic assessment of mitochondrial flux, spatial organization, and functional heterogeneity within osteoblasts, osteoclasts, osteocytes, and the surrounding bone microenvironment.

## 5. Therapeutic Strategies Targeting Mitochondrial Dynamics

Mitochondrial dynamics has emerged as an attractive therapeutic target for DM and its complications. Accumulating evidence indicates that pharmacological modulation of mitochondrial fission, fusion, and mitophagy can restore mitochondrial quality control, alleviate oxidative stress, suppress inflammation, and improve cellular bioenergetics [153]. Several clinically approved drugs and experimental compounds have demonstrated beneficial effects by targeting key regulators of mitochondrial dynamics, highlighting the translational potential of mitochondria-directed therapies. As illustrated in Figure 3, metformin, sodium–glucose cotransporter 2 (SGLT2) inhibitors, mitochondrial fission inhibitors, and mitochondria-targeted antioxidants represent four major therapeutic strategies that restore mitochondrial homeostasis and mitigate the progression of diabetes and its complications.

### 5.1. Metformin

Beyond its glucose-lowering effect, metformin has emerged as a clinically established modulator of cellular energy metabolism and mitochondrial homeostasis. Metformin can inhibit mitochondrial respiratory-chain activity at pharmacologically relevant concentrations, thereby altering cellular energy status and activating AMPK-dependent metabolic signaling [154]. Downstream activation of AMPK and SIRT1/PGC-1α-associated pathways has been linked to improved mitochondrial biogenesis, redox homeostasis, and mitochondrial quality control. Importantly, however, the effects of metformin on mitochondrial dynamics are context- and dose-dependent rather than uniformly directed toward inhibition of mitochondrial fission [155]. In hepatocytes, metformin has been shown to activate AMPK-dependent phosphorylation of MFF, promoting DRP1 recruitment to mitochondria and increasing mitochondrial fission at experimentally relevant concentrations of approximately 50–75 μM [156]. In this context, the increased fission was associated with enhanced mitochondrial respiration and maintenance of mitochondrial number, and was proposed to facilitate the removal of damaged mitochondria through mitophagy [156]. Importantly, regulated mitochondrial fission should be distinguished from excessive or sustained mitochondrial fragmentation. Fission is a normal component of mitochondrial quality control that enables the segregation of damaged mitochondrial components and can facilitate their subsequent elimination by mitophagy, whereas excessive or dysregulated fission may promote mitochondrial dysfunction and cellular injury [135,157]. Therefore, metformin can be reconciled by considering its potential role in restoring appropriately regulated mitochondrial turnover rather than simply increasing or suppressing fission. In this context, the beneficial effect of metformin may reflect improved coordination among fission, mitophagy, fusion, and mitochondrial biogenesis, thereby maintaining mitochondrial quality control and metabolic function.

The clinical relevance of metformin is considerably stronger than that of most mitochondria-targeted compounds discussed in this review. Metformin has been used clinically for decades and remains a first-line pharmacological treatment for type 2 diabetes. In the UK Prospective Diabetes Study, long-term metformin treatment in overweight patients with newly diagnosed type 2 diabetes reduced diabetes-related endpoints and all-cause mortality compared with conventional treatment, providing robust evidence for its clinical efficacy. However, these clinical benefits should not be interpreted as direct clinical validation of mitochondrial-dynamics modulation. Most mechanistic evidence linking metformin to mitochondrial fission, fusion, mitophagy, or biogenesis has been derived from cellular and animal models, whereas clinical trials have primarily evaluated glycemic control, cardiovascular outcomes, metabolic parameters, or disease progression. Thus, whether modulation of mitochondrial dynamics represents a major mediator of metformin’s clinical benefits in humans remains incompletely established.

An additional consideration is the substantial difference between experimental exposure and clinically achievable drug concentrations. After oral administration, metformin is absorbed from the gastrointestinal tract and enters the portal circulation, with substantial tissue accumulation, particularly in the intestine, liver, and kidney [158,159]. Although no universally accepted therapeutic concentration range has been established for metformin [155], clinical pharmacokinetic studies indicate that systemic plasma concentrations in patients receiving standard metformin treatment are generally in the low micromolar range, approximately 10–40 μM [158]. Portal venous and hepatic exposure is expected to be higher because of intestinal absorption and first-pass hepatic uptake; however, direct measurements of metformin concentrations in these compartments in humans remain limited. Based on available human plasma concentrations and pharmacokinetic considerations, hepatic exposure has been estimated to reach approximately 60–90 μM [158]. Animal studies further demonstrate substantial tissue accumulation and higher portal venous than systemic concentrations following oral administration [159]. Notably, many in vitro studies investigating the mitochondrial actions of metformin employ concentrations ranging from 0.5 to 10 mM, and concentrations above 1 mM are considered suprapharmacological and do not occur in the clinical setting [158,160]. Accordingly, mitochondrial effects observed at millimolar concentrations, particularly direct inhibition of respiratory-chain complex I, should be interpreted cautiously and should not be assumed to reflect mitochondrial responses occurring at clinically relevant exposure levels. Thus, experimental studies using concentrations in the low micromolar to low hundreds of micromolar range may provide greater translational relevance, although tissue-specific accumulation, transporter expression, exposure duration, and experimental conditions should still be considered.

The safety profile and therapeutic window of metformin further illustrate the challenges of mitochondrial pharmacology. Because inhibition of mitochondrial respiration can increase lactate production under certain conditions, excessive systemic exposure, particularly in the setting of impaired renal clearance or other conditions that compromise lactate metabolism, may increase the risk of metformin-associated lactic acidosis [161]. Long-term treatment has also been associated with vitamin B12 deficiency, emphasizing that modulation of cellular metabolism can have consequences beyond the intended mitochondrial pathway [162]. These limitations do not negate the clinical utility of metformin, but they highlight an important translational principle: mitochondrial modulation is beneficial only within an appropriate pharmacological and metabolic context. Accordingly, strategies that produce strong mitochondrial inhibition in experimental models should not automatically be considered therapeutically advantageous.

Combination therapy may represent a more rational strategy for simultaneously addressing systemic metabolic abnormalities and mitochondrial dysfunction than indiscriminate modulation of a single mitochondrial pathway. Clinically, metformin is frequently combined with glucagon-like peptide-1 receptor agonists (GLP-1RAs) in patients with type 2 diabetes. A network meta-analysis of 34 randomized controlled trials involving 12,993 patients with inadequate glycemic control on metformin showed that adding GLP-1RAs consistently improved glycemic control and reduced body weight, without increasing the incidence of hypoglycemia; tirzepatide and semaglutide showed particularly pronounced effects on HbA1c and body weight [163]. These findings support the clinical value of combining metformin with agents that act through complementary metabolic pathways. However, this clinical benefit should not be equated with a confirmed mitochondria-targeted synergistic effect. The available clinical trials primarily evaluated glycemic and weight-related outcomes rather than mitochondrial fission, mitophagy, or mitochondrial biogenesis. Thus, whether the metabolic benefits of metformin–GLP-1RA combination therapy are partly mediated by coordinated improvement of mitochondrial quality control remains to be determined. This distinction is important because combination therapy may improve systemic metabolic stress while simultaneously modifying mitochondrial homeostasis, but excessive or overlapping metabolic effects could also alter cellular energy balance in a tissue- and disease-dependent manner. Future studies should therefore determine whether the clinical benefits of such combinations are associated with measurable changes in mitochondrial dynamics and mitophagy at pharmacologically relevant tissue exposures. Taken together, metformin represents an instructive example of the distinction between a clinically validated metabolic drug and a clinically validated mitochondrial-dynamics therapy. Its extensive clinical use, established safety profile, and evidence for long-term metabolic benefit make it an attractive candidate for mitochondrial repurposing. Nevertheless, the precise contribution of mitochondrial dynamics to its therapeutic efficacy remains unresolved, and its effects on fission and mitophagy appear to depend on tissue, concentration, and metabolic state. Future studies should therefore integrate pharmacokinetic exposure, tissue-specific mitochondrial phenotypes, and dynamic measurements of fission, fusion, mitophagy flux, and mitochondrial biogenesis. Such approaches may help determine whether metformin can be rationally combined with tissue-specific or mitochondria-targeted interventions to achieve more precise therapeutic modulation of mitochondrial homeostasis in diabetes and its complications.

### 5.2. Sodium–Glucose Cotransporter 2 Inhibitors

Sodium–glucose cotransporter 2 inhibitors (SGLT2is), including empagliflozin, dapagliflozin, and canagliflozin, have emerged as an important class of antidiabetic agents with cardiovascular and renal benefits that extend beyond glucose lowering. Large cardiovascular and renal outcome trials have demonstrated that SGLT2is reduce hospitalization for heart failure and slow the progression of diabetic kidney disease, including reductions in albuminuria, worsening kidney function, and kidney failure [164]. In patients with type 2 diabetes and established cardiovascular disease or chronic kidney disease, these agents also reduce major cardiovascular events and/or cardiovascular death, highlighting their therapeutic relevance to major diabetic complications rather than glycemic control alone [165]. Importantly, these cardiorenal benefits are only partly explained by improvements in glycemic control, suggesting that mechanisms involving hemodynamic regulation, substrate utilization, inflammation, oxidative stress, and mitochondrial homeostasis may contribute to their organ-protective effects. However, the precise contribution of mitochondrial mechanisms to the clinical benefits of SGLT2is remains incompletely defined.

Preclinical studies indicate that SGLT2is can modulate several components of mitochondrial quality control in a tissue-dependent manner. In diabetic renal tubular cells, empagliflozin attenuated high-glucose-induced mitochondrial fragmentation by normalizing the expression of fission- and fusion-related proteins, including DRP1, FIS1, MFN1, and MFN2, while simultaneously enhancing mitochondrial biogenesis and autophagic activity [166]. More recent studies have provided additional mechanistic evidence in DN. Dapagliflozin activated the SIRT1–PINK1–Parkin pathway and enhanced mitophagy, accompanied by restoration of mitochondrial membrane potential and reduction in mitochondrial ROS, suggesting that restoration of mitochondrial quality control may contribute to its renoprotective effects [167]. In DCM, empagliflozin has also been shown to improve mitochondrial function and ATP production while reducing mitochondrial damage and oxidative stress, indicating that its effects may extend beyond the kidney to metabolically demanding organs [168].

The translational relevance of these mechanisms is supported, but not yet established, by human studies. In patients with type 2 diabetes and albuminuria, dapagliflozin altered urinary metabolites related to mitochondrial metabolism, including pathways involved in the tricarboxylic acid cycle, glycine degradation, and carnitine biosynthesis [169]. Empagliflozin treatment has also been associated with changes in urinary mtDNA and IL-1β in patients with type 2 diabetes, suggesting an improvement in renal mitochondrial stress and inflammatory responses [170]. However, clinical evidence is not uniformly consistent across tissues. A controlled study of patients with type 2 diabetes found that five weeks of dapagliflozin treatment did not significantly alter skeletal-muscle mitochondrial function or mitochondrial network integrity, although substrate utilization shifted toward fatty acid and ketone-body metabolism [171]. Importantly, this metabolic shift may provide a mechanistic link between SGLT2 inhibition and mitochondrial energy metabolism. By promoting urinary glucose loss and lowering the insulin-to-glucagon ratio, SGLT2 inhibition can enhance lipolysis and hepatic ketogenesis, increasing circulating ketone bodies, particularly β-hydroxybutyrate [172]. Ketone bodies can be taken up by oxidative tissues and converted to acetyl-CoA for entry into the tricarboxylic acid cycle, thereby providing an alternative substrate for mitochondrial ATP production. In the diabetic heart, empagliflozin has been shown to enhance ketone-body utilization and improve myocardial ATP production while reducing mitochondrial dysfunction and oxidative stress [168]. These findings suggest that enhanced ketone-body availability and utilization may contribute to the mitochondrial effects of SGLT2is, although the extent to which this pathway directly mediates their clinical organ-protective effects remains to be established. Overall, mitochondrial effects of SGLT2is appear to be strongly tissue- and metabolic-context-dependent and should not be generalized from one organ to another.

Pharmacokinetic and target-distribution considerations provide an additional limitation. SGLT2 is predominantly expressed in the proximal tubule, and the primary pharmacological action of SGLT2is occurs in the kidney [173]. Accordingly, renal exposure and tubular effects are likely to be particularly relevant to their mitochondrial actions. However, many reported mitochondrial effects in the heart, liver, skeletal muscle, or other tissues may arise indirectly through altered substrate availability, hemodynamics, ketone-body metabolism, or systemic metabolic remodeling rather than direct mitochondrial targeting [174]. This distinction is important because SGLT2is should not be considered conventional mitochondria-targeted drugs in the strict pharmacological sense.

The clinical safety profile further highlights the importance of therapeutic context. Although SGLT2is provide substantial cardiovascular and renal benefits, they increase the risk of genital mycotic infections, volume depletion, and diabetic ketoacidosis (DKA) [175]. A network meta-analysis of 85,997 participants confirmed an increased risk of DKA with SGLT2is, although the absolute incidence remained low [176]. These risks emphasize that the metabolic effects of SGLT2is may have different consequences depending on hydration status, insulin availability, renal function, and disease context, underscoring the importance of an appropriate therapeutic window.

Overall, SGLT2is provide a clinically established example of how modulation of systemic metabolism can be accompanied by substantial improvements in mitochondrial homeostasis. Nevertheless, current evidence does not establish mitochondrial dynamics as a primary clinical target of SGLT2is, and the relative contribution of altered fission–fusion balance, mitophagy, mitochondrial biogenesis, ketone-body metabolism, and hemodynamic effects remains unresolved. Future studies should therefore integrate tissue-specific mitochondrial phenotyping with pharmacokinetic exposure, dynamic assessment of mitophagy, and clinically relevant cardiovascular and renal endpoints to determine whether mitochondrial remodeling is a causal mediator or a downstream consequence of SGLT2 inhibitor therapy.

### 5.3. Mitochondrial Fission Inhibitors

Pharmacological inhibition of excessive mitochondrial fission has emerged as a promising preclinical strategy for diabetic complications. Mdivi-1 was initially reported as a DRP1 inhibitor and has been widely used to suppress pathological mitochondrial fragmentation, with beneficial effects on mitochondrial membrane potential, oxidative stress, and apoptosis in experimental models [177]. However, its translational potential is limited by concerns regarding target specificity. Mdivi-1 has been shown to inhibit mitochondrial complex I at concentrations commonly used to study DRP1-dependent fission, with effects that can occur independently of DRP1, raising concerns that some of its reported protective effects may reflect off-target modulation of mitochondrial respiration rather than selective inhibition of fission [178].

P110 represents a more selective approach because it interferes with the pathological DRP1–FIS1 interaction while largely preserving basal DRP1-dependent mitochondrial fission [179]. Importantly, recent evidence directly supports its potential relevance to diabetic complications: in experimental DN, P110 reduced mitochondrial fragmentation and attenuated renal fibrosis, inflammation, and podocyte injury without lowering blood glucose, suggesting that modulation of mitochondrial dynamics may provide organ-protective effects independently of glycemic control [180]. Nevertheless, these findings remain preclinical, and the peptide nature of P110 presents pharmacokinetic and delivery challenges that must be resolved before clinical translation. Thus, although selective inhibition of pathological rather than physiological mitochondrial fission is an attractive therapeutic concept, further studies are required to establish its tissue distribution, therapeutic window, long-term safety, and clinical efficacy.

### 5.4. Mitochondria-Targeted Antioxidants

Because excessive mitochondrial ROS contributes to oxidative damage and inflammation in diabetes, mitochondria-targeted antioxidants have attracted considerable attention as potential therapeutic agents. MitoQ and SS-31 (elamipretide) use distinct mitochondrial-targeting strategies to enrich their effects within mitochondria and reduce oxidative stress. In experimental diabetic models, MitoQ and SS-31 have been shown to reduce mitochondrial ROS and improve mitochondrial function [181], while MitoQ can additionally attenuate endoplasmic reticulum stress and inflammatory signaling in pancreatic β-cells [180].

Importantly, the translational evidence is more limited than the preclinical literature suggests. In patients with type 2 diabetes, ex vivo treatment of leukocytes with MitoQ reduced mitochondrial ROS and inflammatory markers [182], whereas SS-31 similarly reduced mitochondrial ROS and increased mitochondrial membrane potential and glutathione content while attenuating inflammatory responses [183]. However, these studies primarily evaluated surrogate cellular endpoints in isolated leukocytes rather than direct effects on diabetic target organs or established clinical complications. Thus, improvement in mitochondrial oxidative stress at the cellular level does not necessarily translate into reversal of chronic tissue injury or improvement in organ-level outcomes. This discrepancy may partly reflect the complex and multifactorial nature of diabetic complications, in which mitochondrial oxidative stress represents only one component of a broader pathological network involving metabolic dysfunction, inflammation, vascular abnormalities, and tissue remodeling. In addition, the available clinical studies of mitochondria-targeted antioxidants have generally been limited in duration and heterogeneous in population, intervention, and outcome measures, making it difficult to determine whether short-term mitochondrial target engagement can produce sustained clinical benefit. Consistent with these limitations, a systematic review and meta-analysis of 19 randomized clinical trials involving 884 participants found limited evidence for beneficial effects of mitochondria-targeted antioxidants on glycemic control, cardiovascular outcomes, or oxidative-stress-related endpoints, with the certainty of evidence generally low or very low [184].

Differences in drug distribution and pharmacokinetics also represent important barriers to translation. For example, the oral bioavailability and tissue exposure of MitoQ are influenced by intestinal transport and metabolism, and experimental studies have shown that MitoQ undergoes intracellular reduction and subsequent conjugation, while intestinal transport can be influenced by efflux transporters such as P-glycoprotein and breast cancer resistance protein [185], and mitochondrial accumulation does not necessarily ensure equivalent exposure across organs [184]. In addition, the clinical development of SS-31 has demonstrated that favorable mitochondrial effects observed experimentally do not necessarily translate into improvements in organ-level clinical outcomes. In a phase II trial of patients with heart failure with reduced ejection fraction, elamipretide was well tolerated but did not significantly improve left ventricular end-systolic volume or ejection fraction after 28 days of treatment [186]. Therefore, differences in tissue distribution, mitochondrial target engagement, and disease-specific mitochondrial dysfunction may partly explain why favorable biochemical effects observed in experimental systems do not necessarily translate into consistent clinical benefits.

Thus, mitochondria-targeted antioxidants should not be regarded simply as more potent conventional antioxidants. Their therapeutic efficacy is likely to depend on mitochondrial accumulation, tissue-specific exposure, disease stage, and the magnitude and source of mitochondrial ROS. Moreover, current clinical evidence does not establish MitoQ or SS-31 as effective treatments for diabetic complications. Future studies should therefore integrate pharmacokinetic profiling, tissue-specific mitochondrial target engagement, and clinically meaningful organ-level outcomes to determine whether suppression of mitochondrial ROS can be translated into durable therapeutic benefit.

Collectively, current pharmacological approaches target different aspects of mitochondrial homeostasis, including mitochondrial fission, mitophagy, oxidative stress, and metabolic remodeling. However, their therapeutic evidence varies substantially across disease models and levels of clinical validation. Table 2 summarizes representative therapeutic strategies, their molecular targets, effects on mitochondrial homeostasis, relevant diabetic complications, evidence levels, human clinical status, and major translational limitations. This overview highlights the distinction between experimentally promising mitochondrial modulators and therapies with established clinical applicability.

### 5.5. Challenges, Limitations, and Emerging Therapeutic Perspectives

Despite substantial progress in targeting mitochondrial dynamics and quality control, several barriers remain before these strategies can be translated into clinically effective therapies for diabetes and its complications. First, the evidence base remains predominantly preclinical. Although metformin and SGLT2 inhibitors have established clinical indications and are associated with mitochondrial effects, direct clinical evidence linking modulation of mitochondrial dynamics to improved diabetic complications remains limited. Similarly, emerging agents such as P110 and mitochondria-targeted antioxidants have shown encouraging results in experimental models, but their clinical efficacy has not yet been established.

Second, mitochondrial dynamics are highly tissue- and context-dependent. Excessive fission may promote mitochondrial damage in one pathological setting, whereas fission is also required for mitochondrial turnover and quality control under physiological conditions. Therefore, indiscriminate inhibition of fission or persistent activation of mitophagy may impair rather than restore mitochondrial homeostasis. This context dependence is also evident clinically, as mitochondrial responses to pharmacological interventions may differ substantially among the kidney, heart, skeletal muscle, liver, and pancreatic β-cells. Therapeutic strategies should therefore aim to restore an appropriate range of mitochondrial dynamics rather than simply increase or decrease a single pathway.

Third, pharmacokinetic properties, tissue distribution, and drug specificity remain major translational challenges. Systemically administered drugs may produce markedly different exposures across organs, while mitochondria-targeted compounds may not achieve sufficient concentrations at the relevant disease site. Potential off-target effects and exposure-dependent biological responses may complicate the clinical translation of mitochondrial modulators. Mdivi-1 illustrates the challenge of limited target specificity, whereas metformin highlights how mitochondrial effects can vary substantially with drug exposure. For peptide- or mitochondria-targeted agents, stability, bioavailability, tissue penetration, and drug-delivery efficiency represent additional considerations. For therapies intended to influence central metabolic or neurovascular processes, blood–brain barrier penetration represents an additional determinant of target engagement and may substantially restrict the translational applicability of systemically administered mitochondrial modulators.

Fourth, mitochondrial target engagement does not necessarily translate into clinical benefit. Improvements in mitochondrial ROS, membrane potential, mitophagy, or mitochondrial morphology observed in cells or animal models may represent intermediate biological responses rather than causal determinants of organ-level recovery. The limited or heterogeneous results of human studies with mitochondria-targeted antioxidants further emphasize this issue. Future clinical trials should therefore incorporate validated biomarkers of mitochondrial target engagement together with clinically meaningful outcomes, rather than relying exclusively on surrogate molecular endpoints.

Fifth, Biological sex may represent an important yet understudied determinant of mitochondrial dynamics in diabetes and its complications. Sex-related differences in mitochondrial metabolism, oxidative stress responses, and mitochondrial quality control may influence disease phenotypes as well as responses to mitochondria-directed interventions. Future studies should therefore consider biological sex as a potential modifier of mitochondrial phenotypes and therapeutic efficacy, particularly in translational and clinical research.

Finally, future therapeutic development should move toward disease- and tissue-specific precision modulation. Combination strategies integrating metabolic control with selective correction of mitochondrial dysfunction may be more effective than excessive manipulation of a single mitochondrial pathway. Recent work has highlighted the potential of combining mitochondrial quality-control modulation with metabolic interventions and exploiting emerging approaches such as targeted drug delivery, mitochondrial biomarkers, omics-based patient stratification, and precision medicine. However, these approaches remain exploratory, and their clinical value will depend on establishing appropriate therapeutic windows, identifying patients most likely to benefit, and demonstrating sustained organ protection without disrupting physiological mitochondrial turnover. Thus, the future of mitochondria-targeted therapy in diabetes is likely to depend not on maximal modulation of mitochondrial pathways, but on precise, tissue-specific restoration of mitochondrial homeostasis.

## 6. Conclusions and Future Perspectives

Mitochondrial dynamics has emerged as a fundamental regulator of mitochondrial quality control and metabolic homeostasis in DM and its complications. Rather than functioning as isolated processes, mitochondrial fission, fusion, and mitophagy constitute an interconnected regulatory network that preserves mitochondrial integrity through continuous remodeling, selective elimination of damaged organelles, and maintenance of bioenergetic capacity. Accumulating evidence indicates that disruption of this dynamic equilibrium contributes to pancreatic β-cell dysfunction, insulin resistance, and the progression of DN, DCM, DR, and DOP. However, an important concept emphasized throughout this review is that alterations in mitochondrial morphology should not be universally interpreted as primary pathogenic events. Depending on the metabolic context, mitochondrial fragmentation or hyperfusion may represent adaptive responses, compensatory mechanisms, or downstream consequences of cellular stress, highlighting the context-dependent nature of mitochondrial dynamics.

Increasing mechanistic evidence further demonstrates that mitochondrial dynamics is closely integrated with endoplasmic reticulum–mitochondria communication, oxidative stress, inflammatory signaling, and mitochondrial quality control. These interactions suggest that therapeutic efficacy is more likely to be achieved by restoring mitochondrial homeostasis than by indiscriminately promoting either mitochondrial fusion or fission. Accordingly, pharmacological agents including metformin, sodium–glucose cotransporter 2 inhibitors, mitochondrial fission inhibitors, and mitochondria-targeted antioxidants have shown considerable promise in experimental models by improving mitochondrial function, suppressing oxidative damage, and enhancing mitophagy. Nevertheless, the translation of these findings into clinical practice remains limited by challenges including tissue-specific responses, off-target effects, inadequate mitochondrial delivery, and the incomplete understanding of long-term modulation of mitochondrial dynamics.

Future research should focus on several key directions. First, greater attention should be given to the tissue-specific and stage-dependent roles of mitochondrial dynamics, as identical molecular pathways may exert divergent effects in different diabetic organs. Second, elucidating the molecular crosstalk among mitochondrial dynamics, mitophagy, and endoplasmic reticulum stress will facilitate the identification of more precise therapeutic targets. Third, the development of organ-targeted mitochondrial delivery systems and combination therapeutic strategies may improve both efficacy and safety. Finally, the integration of mitochondrial imaging, multi-omics technologies, and circulating mitochondrial biomarkers may provide novel opportunities for early disease stratification and personalized intervention. Collectively, maintaining the physiological balance among mitochondrial fission, fusion, and mitophagy represents a promising strategy for preventing and treating diabetes and its diverse complications.

## Figures and Tables

**Figure 1:**
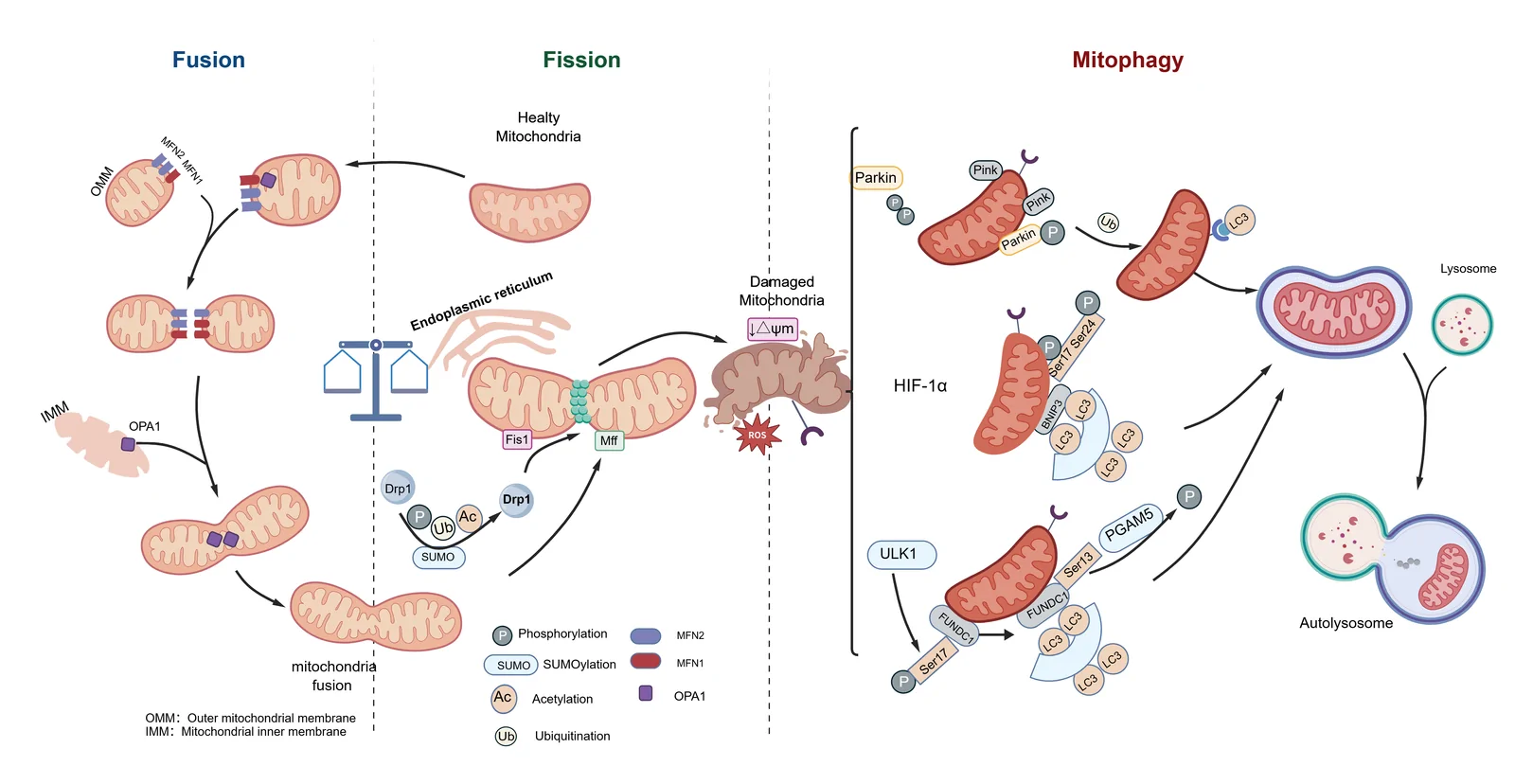
Molecular mechanisms of mitochondrial fusion, fission, and mitophagy.

**Figure 2:**
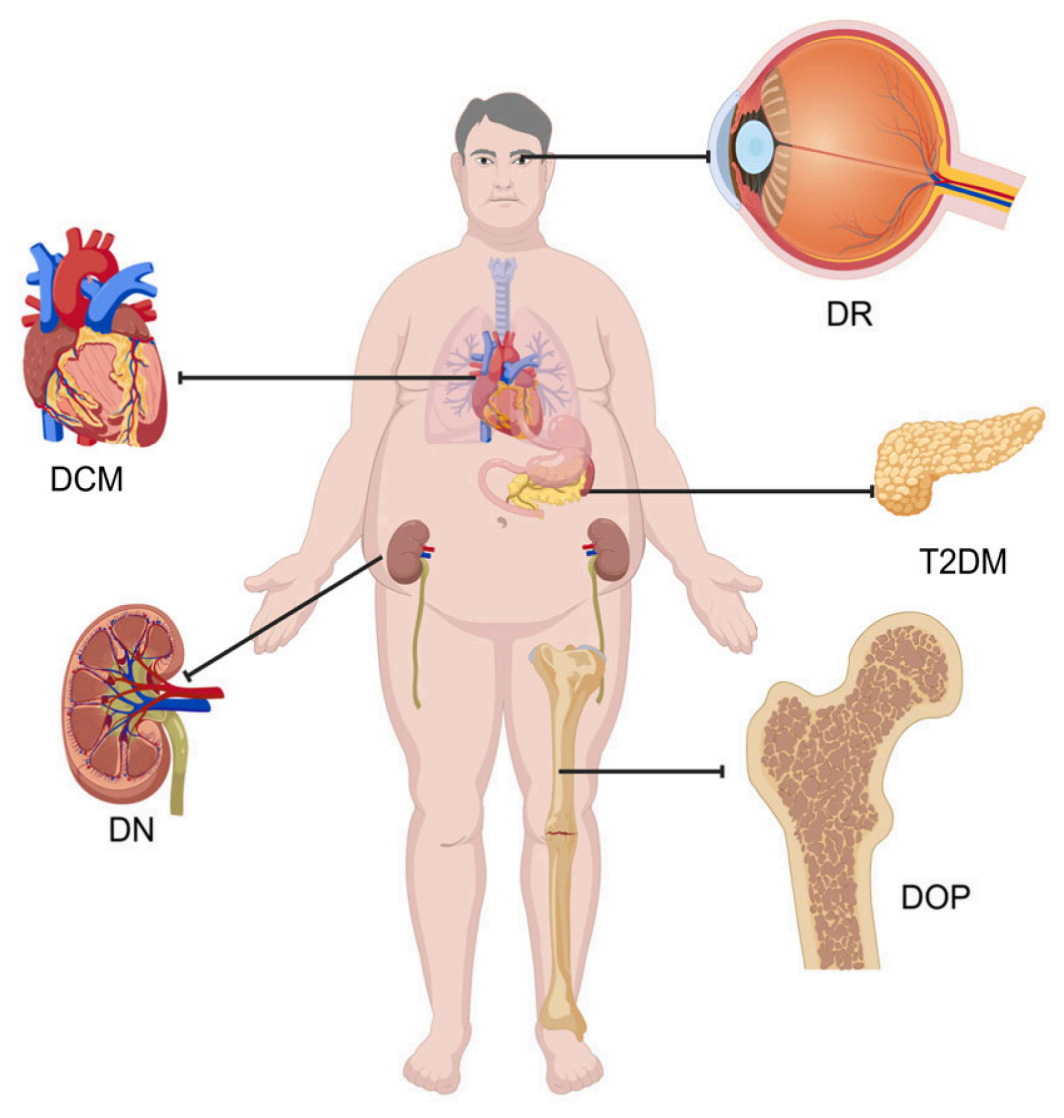
Diabetes mellitus and its major complications. T2DM: Type 2 diabetes mellitus. DN: Diabetic nephropathy. DCM: Diabetic cardiomyopathy. DR: Diabetic retinopathy. DOP: Diabetic osteoporosis.

**Figure 3:**
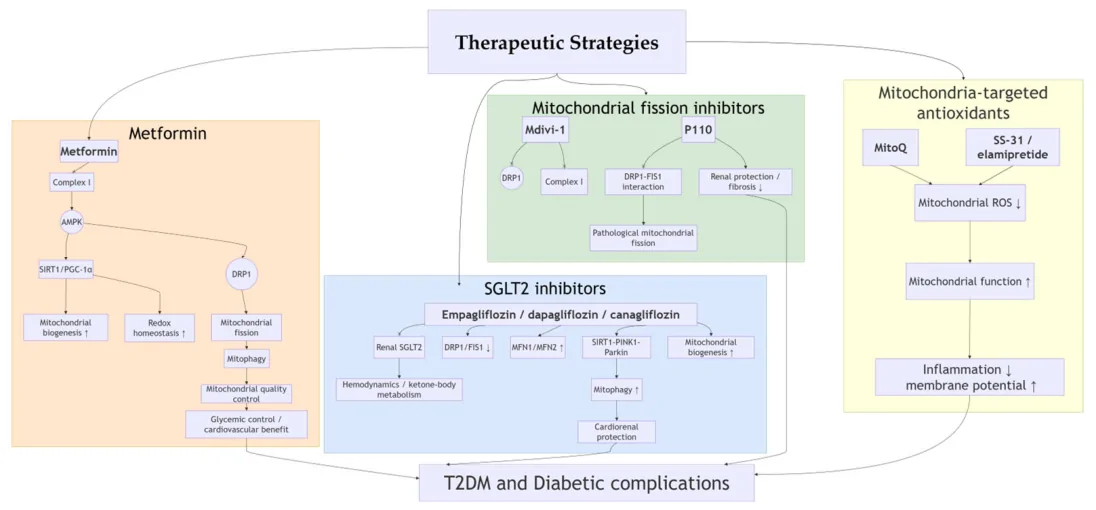
Therapeutic strategies targeting mitochondrial dynamics in diabetes and its complications. Metformin activates AMPK to promote mitochondrial biogenesis, redox balance and mitophagy while restraining DRP1-dependent mitochondrial fission. Mitochondrial fission inhibitors (Mdivi-1, P110) block pathological mitochondrial fragmentation via DRP1 suppression. SGLT2 inhibitors (empagliflozin, dapagliflozin, canagliflozin) improve mitochondrial dynamics and mitophagy for cardiorenal protection. Mitochondria-targeted antioxidants (MitoQ, SS-31/elamipretide) reduce mitochondrial ROS, recover mitochondrial membrane potential and relieve inflammation. These interventions collectively ameliorate T2DM and diabetic complications. **↑** indicates upregulation or activation; **↓** indicates downregulation or inhibition.

**Table 1:** Post-Translational Modifications Regulating Mitochondrial Dynamics and Mitophagy.

Process	Key Regulator	Modification	Site(s)	Functional Consequence
Mitochondrial Fission	DRP1	Phosphorylation	Ser616	Promotes mitochondrial recruitment and fission
	DRP1	Phosphorylation	Ser637	Generally suppresses mitochondrial recruitment and fission
	DRP1	Acetylation	Lys642	Promotes Ser616 phosphorylation, DRP1 oligomerization, mitochondrial translocation, and fission
	MFF	Phosphorylation	Ser172	Promotes DRP1 recruitment to mitochondria
	MARCH5/ MiD49	Ubiquitination	—	Regulates MiD49 turnover and DRP1-dependent fission
Mitochondrial Fusion	MFN1	—	—	Promotes outer mitochondrial membrane fusion
	MFN2	Phosphorylation	Ser442	Regulates MFN2-mediated fusion and ER–mitochondria communication
	OPA1	—	—	Maintains inner-membrane fusion and cristae architecture
Mitophagy	PINK1	Autophosphorylation	Ser228	Promotes PINK1 activation and downstream signaling
	Parkin	Phosphorylation	Ser65	Activates Parkin ubiquitin ligase activity
	Ubiquitin	Phosphorylation	Ser65	Amplifies Parkin activation and mitochondrial recruitment
	BNIP3	Phosphorylation	Ser17	Enhances LC3 binding and mitophagy receptor activity
	FUNDC1	Phosphorylation	Ser17	Promotes LC3 binding and mitophagy
	FUNDC1	Phosphorylation	Ser13	Reduces LC3 binding; dephosphorylation promotes mitophagy
	OPTN	Phosphorylation	Ser177	Enhances mitochondrial recruitment and selective mitophagy

(—) Unknown or not described.

**Table 2:** Representative therapeutic strategies targeting mitochondrial dynamics and quality control in diabetes and its complications. ↓: indicates downregulation or inhibition.

Therapeutic Agent	Primary Target/Pathway	Mitochondrial Effect	Diabetic Complication	Evidence Level	Human Clinical Evidence	Major Translational Limitation
Metformin	AMPK/DRP1/MFF	Context-dependent fission/mitophagy	T2DM, DN, cardiovascular complications	Clinical + preclinical	Yes	Concentration, tissue exposure, MALA/B12
SGLT2i	SGLT2/metabolic remodeling	Dynamics, mitophagy, biogenesis	DN, DCM, cardiovascular complications	Clinical + preclinical	Yes	Tissue dependence, DKA
Mdivi-1	DRP1	↓ pathological fission	Experimental diabetic complications	Preclinical	No	Complex I/off-target effects
P110	DRP1–FIS1	Selective inhibition of pathological fission	DN	Preclinical	No	Peptide PK/delivery
MitoQ	Mitochondrial ROS	↓ mtROS	Diabetes-related metabolic injury	Preclinical + human exploratory	Yes, early	Tissue exposure/clinical efficacy
SS-31/ Elamipretide	Cardiolipin	Mitochondrial membrane stabilization	Diabetes-related mitochondrial dysfunction	Preclinical + human	Yes, clinical trials	Clinical efficacy/PK

## Data Availability

No new data were created or analyzed in this study. Data sharing is not applicable to this article.
